# Activities and Structure-Function Analysis of Fission Yeast Inositol Pyrophosphate (IPP) Kinase-Pyrophosphatase Asp1 and Its Impact on Regulation of *pho1* Gene Expression

**DOI:** 10.1128/mbio.01034-22

**Published:** 2022-05-10

**Authors:** Bradley Benjamin, Angad Garg, Nikolaus Jork, Henning J. Jessen, Beate Schwer, Stewart Shuman

**Affiliations:** a Molecular Biology Program, Memorial Sloan Kettering Cancer Center, New York, New York, USA; b Gerstner Sloan Kettering Graduate School of Biomedical Sciences, New York, New York, USA; c Institute of Organic Chemistry, University of Freiburg, Freiburg, Germany; d Center for Biological Signaling Studies, University of Freiburg, Freiburg, Germany; e Spemann Graduate School of Biology and Medicine, University of Freiburg, Freiburg, Germany; f Dept. of Microbiology and Immunology, Weill Cornell Medical College, New York, New York, USA; Harvard Medical School

**Keywords:** Asp1, fission yeast, inositol polyphosphate kinase, inositol pyrophosphates

## Abstract

Inositol pyrophosphates (IPPs) are signaling molecules that regulate cellular phosphate homeostasis in diverse eukaryal taxa. In fission yeast, mutations that increase 1,5-IP_8_ derepress the *PHO* regulon while mutations that ablate IP_8_ synthesis are *PHO* hyper-repressive. Fission yeast Asp1, the principal agent of 1,5-IP_8_ dynamics, is a bifunctional enzyme composed of an N-terminal IPP kinase domain and a C-terminal IPP pyrophosphatase domain. Here we conducted a biochemical characterization and mutational analysis of the autonomous Asp1 kinase domain (aa 1–385). Reaction of Asp1 kinase with IP_6_ and ATP resulted in both IP_6_ phosphorylation to 1-IP_7_ and hydrolysis of the ATP γ-phosphate, with near-equal partitioning between productive 1-IP_7_ synthesis and unproductive ATP hydrolysis under optimal kinase conditions. By contrast, reaction of Asp1 kinase with 5-IP_7_ is 22-fold faster than with IP_6_ and is strongly biased in favor of IP_8_ synthesis versus ATP hydrolysis. Alanine scanning identified essential constituents of the active site. We deployed the Ala mutants to show that derepression of *pho1* expression correlated with Asp1’s kinase activity. In the case of full-length Asp1, the activity of the C-terminal pyrophosphatase domain stifled net phosphorylation of the 1-position during reaction of Asp1 with ATP and either IP_6_ or 5-IP_7_. We report that inorganic phosphate is a concentration-dependent enabler of net IP_8_ synthesis by full-length Asp1 *in vitro*, by virtue of its antagonism of IP_8_ turnover.

## INTRODUCTION

Inositol pyrophosphates (IPPs) IP_7_ and IP_8_ are signaling molecules that figure prominently in eukaryal phosphate homeostasis, a transcriptional response to phosphate availability in which genes involved in extracellular phosphate acquisition are upregulated (1–6). IP_8_ is generated from phytic acid (IP_6_) by the sequential action of IPP kinases Kcs1/IP6K, which converts IP_6_ to 5-IP_7_, and Asp1/Vip1/PPIP5K, which converts 5-IP_7_ to 1,5-IP_8_ (7, 8). Asp1, Vip1, and PPIP5K—as they are named in fission yeast, budding yeast, and humans, respectively—are bifunctional enzymes composed of an N-terminal IPP kinase domain that synthesizes IP_8_ and a C-terminal IPP pyrophosphatase domain, of the histidine acid phosphatase enzyme family, that converts IP_8_ back to 5-IP_7_ (9, 10). Asp1/Vip1/PPIP5K can also phosphorylate IP_6_ to yield 1-IP_7_ and de-phosphorylate 1-IP_7_ back to IP_6_. The isolated N-terminal IPP kinase domains of Asp1/Vip1/PPIP5K have autonomous IPP kinase activity (9–13). The Shears laboratory has conducted elegant structural and functional studies of the kinase domain of human PPIP5K isoform 2 (14).

The fission yeast phosphate homeostasis (*PHO*) regulon (15) comprises three phosphate acquisition genes—*pho1* (cell surface acid phosphatase), *pho84* (inorganic phosphate transmembrane transporter), and *tgp1* (glycerophosphate transporter)—that are repressed under phosphate-replete conditions by 5′ flanking lncRNAs *prt*, *prt2*, and *nc-tgp1*, respectively (16). lncRNA transcription across the *PHO* mRNA promoters displaces the activating transcription factor Pho7 from its DNA binding sites (16). The *PHO* regulon is derepressed in phosphate-replete cells by genetic manipulations that favor precocious lncRNA 3′-processing/termination in response to poly(A) signals upstream of the mRNA promoters (17, 18).

*PHO* lncRNA termination is subject to metabolite control by inositol pyrophosphates (19). A pyrophosphatase-defective *asp1-H397A* allele that increases the level of IP_8_ (9,10) derepresses the *PHO* regulon, and prompts precocious termination of *prt* lncRNA synthesis, in a manner dependent on the cleavage and polyadenylation factor complex (CPF) and transcription termination factor Rhn1. An *asp1*Δ null allele that eliminates intracellular IP_8_ and 1-IP_7_ results in *pho1* hyper-repression. Synthetic lethality of *asp1*Δ (no IP_8_) with CPF subunit mutations suggested that IP_8_ (or 1-IP_7_) plays an important role in essential 3′-processing/termination events, albeit in a manner genetically redundant to CPF (19). These results established a novel action for IPPs in cell physiology as agonists of Pol2 transcription termination.

To better understand the role of 1,5-IP_8_ in fission yeast, we set out here to further characterize the fission yeast Asp1 kinase responsible for its synthesis. We report that (i) reaction of purified recombinant Asp1 kinase (aa 1–385) with IP_6_ and [γ^32^P]ATP results in both IP_6_ phosphorylation to ^32^P-IP_7_ and ATP hydrolysis to ^32^P_i_; and (ii) Asp1 kinase hydrolyzes ATP in the absence of IP_6_. The partitioning between “productive” IPP synthesis and “unproductive” ATP hydrolysis is sensitive to reaction conditions (e.g., pH and Mg^2+^ concentration). Reaction of Asp1 kinase with 5-IP_7_ is 22-fold faster than with IP_6_ and is strongly biased in favor of 1,5-IP_8_ formation instead of ATP hydrolysis. A mutational analysis of Asp1 kinase, guided by the structure of the human PPIP5K2 transition-state during conversion of 5-IP_7_ to 1,5-IP_8_ (14), identified essential constituents of the active site. Characterization of purified recombinant full-length Asp1 shows that (i) the activity of the C-terminal pyrophosphatase domain squelches the yield of 1-IPPs during reaction with ATP and IP_6_ or 5-IP_7_; and (ii) pyrophosphatase inactivating mutation H397A restores IPP synthesis. We find that inorganic phosphate is a concentration-dependent activator of net IP_8_ synthesis by full-length Asp1.

## RESULTS

### Purification of recombinant Asp1 kinase.

**FIG 1:**
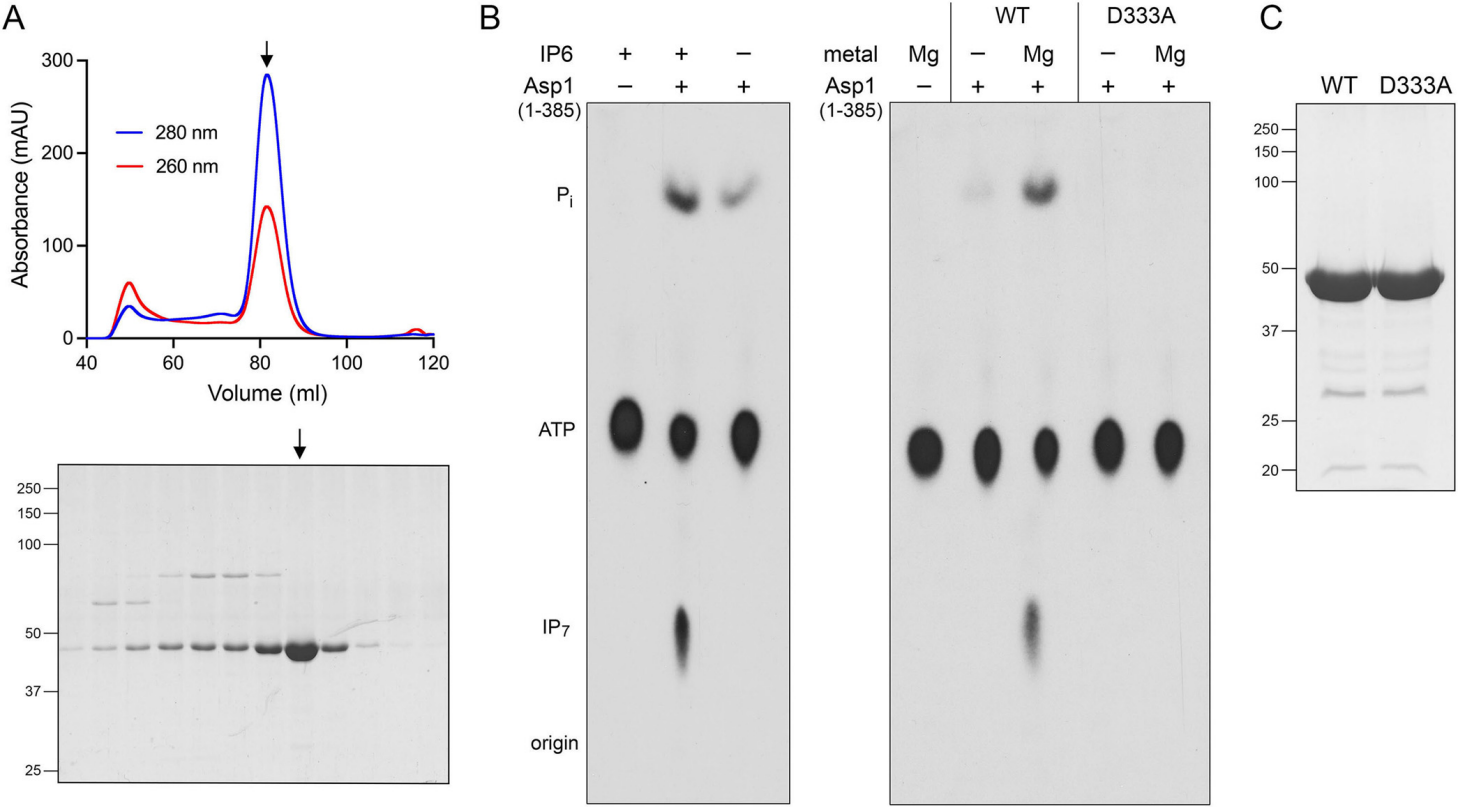
IP_6_ kinase and ATP phosphohydrolase activity of recombinant Asp1-(1-385). (A) Elution profile of wild-type Asp1-(1-385) during Superdex-200 gel filtration. The top panel shows the absorbance at 280 nm (blue trace) and 260 nm (red trace) as a function of elution volume. The *A*_260_ > *A*_280_ peak at 50 mL demarcates the void volume. Aliquots (5 μL) of the fractions spanning and flanking the *A*_280_ peak were analyzed by SDS-PAGE. The Coomassie blue-stained gel is shown. The positions and sizes (kDa) of marker proteins are indicated on the left. The fraction corresponding to the *A*_280_ peak containing purified Asp1-(1-385) is indicated by the vertical arrow. (B) Reaction mixtures (20 μL) containing 30 mM HEPES-NaOH (pH 6.8), 50 mM NaCl, 10 mM MgCl_2_, 0.5 mM [γ^32^P]ATP, 1 mM IP_6_, and 2.5 μM Asp1 kinase were incubated at 37°C for 90 min. Individual reaction components were omitted where indicated by –. The products were resolved by PEI cellulose TLC and visualized by autoradiography. The chromatographic origin and the ^32^P-labeled species corresponding to ATP, IP_7_, and P_i_ are indicated on the left. (C) Aliquots (10 μg) of the peak Superdex-200 fractions of wild-type and D333A mutant Asp1 kinase preparations were analyzed by SDS-PAGE. The Coomassie blue-stained gel is shown with the positions and sizes (kDa) of marker proteins indicated on the left.

We produced the N-terminal kinase domain of Asp1 (aa 1–385) in E. coli as a His_10_Smt3 fusion and isolated the protein from a soluble bacterial extract by adsorption to a Ni-agarose column and elution with imidazole. The His_10_Smt3 was removed by treatment with the Smt3 protease Ulp1 and the tag-free native Asp1-(1-385) protein was separated from the tag during a second round of Ni-affinity chromatography. Final purification was achieved by Superdex-200 gel filtration (Fig. 1A). The elution profile of the 44 kDa kinase polypeptide was consistent with it being a monomer in solution. In parallel, we purified a mutated version of Asp1-(1-385) in which the putative catalytic metal-binding residue Asp333 was changed to alanine (Fig. 1C).

### IP_6_ kinase and ATP phosphohydrolase activity.

In previous studies of human PPIP5K2, kinase activity was assayed quantitatively by incubation of enzyme with cold ATP phosphate donor and ^3^H-labeled IP_6_ or IP_7_ phosphate acceptor substrates, followed by HPLC anion exchange column chromatography of the ^3^H-labeled reaction products and liquid scintillation counting of the fractions (13). A separate assay, employing the malachite green reagent, was implemented to detect PPIP5K2-catalyzed release of inorganic phosphate from cold ATP during the kinase reaction (13). Prior studies of budding yeast Vip1 assayed kinase activity by incubation of enzyme with cold ATP and ^32^P-labeled IP_6_, followed by thin-layer chromatography (TLC) separation of the ^32^P-labeled IP_6_ and IPP reaction products (12). Here we implemented a one-pot assay that simultaneously tracked the IP_6_ kinase and potential ATP phosphohydrolase activities of fission yeast Asp1-(1-385). Recombinant protein was incubated for 90 min with 0.5 mM [γ^32^P]ATP, 1 mM cold IP_6_, and 10 mM Mg^2+^. The reactions were quenched with EDTA, and the radiolabeled products were resolved by polyethyleneimine (PEI)-cellulose TLC (Fig. 1B). The complete reaction resulted in the transfer of the labeled γ-phosphate from ATP to IP_6_ to form a ^32^P-labeled IP_7_ product that migrated more slowly than ATP during TLC, consistent with its greater negative charge. No such product was generated when IP_6_ was omitted (Fig. 1B, left panel). Asp1-(1-385) also effected the hydrolysis of the ATP γ-phosphate to liberate ^32^P_i_, whether or not IP_6_ was included in the reaction (Fig. 1B, left panel). The D333A active site mutation eliminated both IP_6_ phosphorylation and ATP hydrolysis (Fig. 1B, right panel), signifying that both activities are intrinsic to the wild-type Asp1 protein.

### Characterization of IP_6_ kinase and ATPase activities.

**FIG 2:**
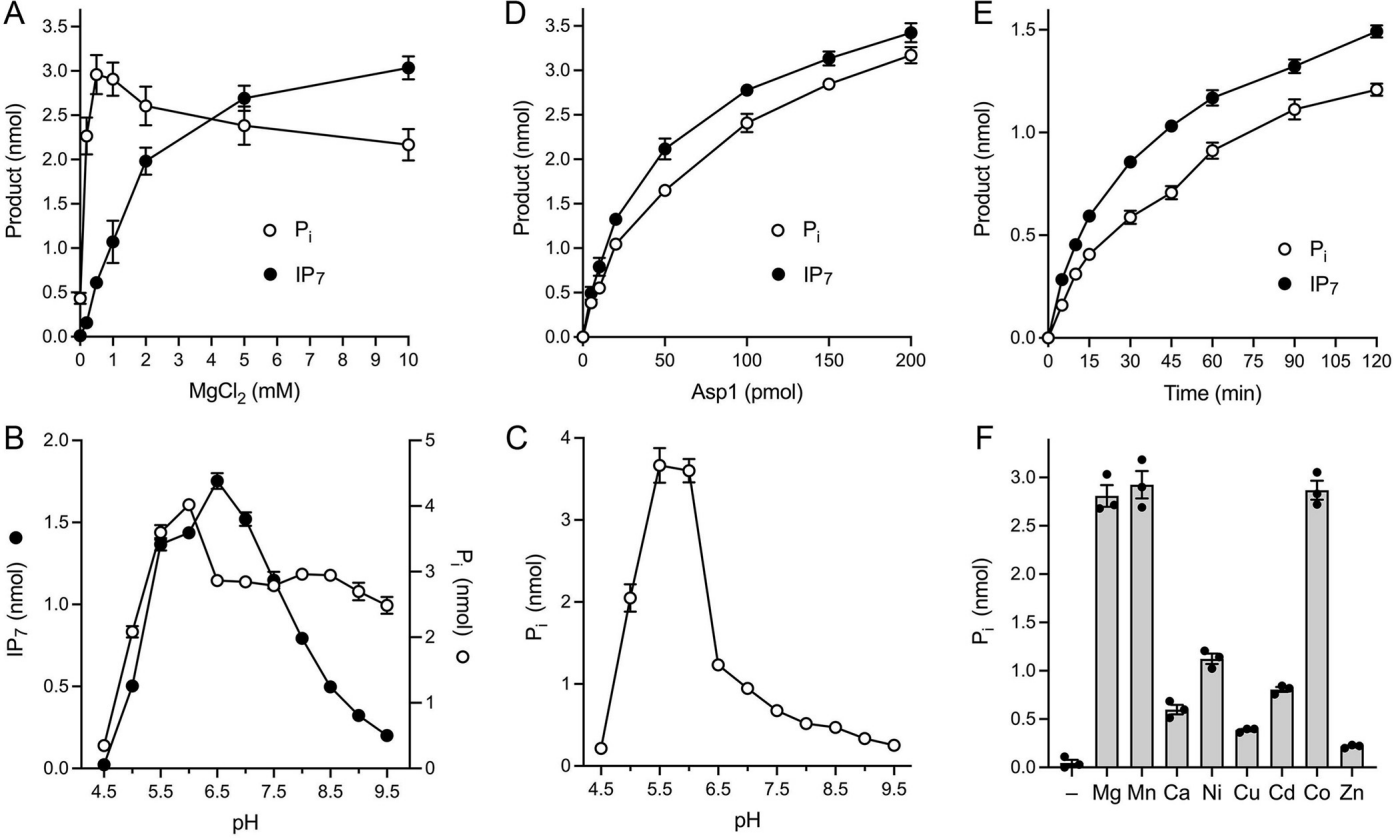
Characterization of the Asp1 IP_6_ kinase and ATPase activities. (A) Magnesium titration. Reaction mixtures (20 μL) containing 30 mM Bis-Tris (pH 6.2), 50 mM NaCl, 0.5 mM (10 nmol) [γ^32^P]ATP, 1 mM (20 nmol) IP_6_, 5 μM (100 pmol) Asp1-(1-385), and magnesium as specified were incubated at 37°C for 90 min. The extents of IP_7_ and P_i_ formation are plotted as a function of magnesium concentration. (B) pH profile. Reaction mixtures (20 μL) containing 30 mM Tris buffer (either Tris-acetate at pH 4.5, 5.0, 5.5, or 6.0; Tris-HCl at pH 6.5, 7.0, 7.5, 8.0, 8.5, 9.0, or 9.5), 50 mM NaCl, 2 mM MgCl_2_, 0.5 mM (10 nmol) [γ^32^P]ATP, 1 mM (20 nmol) IP_6_, and 5 μM (100 pmol) Asp1-(1-385) were incubated at 37°C for 90 min. The extents of IP_7_ and P_i_ formation are plotted as a function of pH. (C) pH profile of ATP hydrolysis in the absence of IP_6_. Reaction mixtures (20 μL) containing 30 mM Tris buffer (either Tris-acetate at pH 4.5, 5.0, 5.5, or 6.0; Tris-HCl at pH 6.5, 7.0, 7.5, 8.0, 8.5, 9.0, or 9.5), 50 mM NaCl, 2 mM MgCl_2_, 0.5 mM (10 nmol) [γ^32^P]ATP, and 5 μM (100 pmol) Asp1-(1-385) were incubated at 37°C for 90 min. The extents of P_i_ formation are plotted as a function of pH. (D) Enzyme titration. Reaction mixtures (20 μL) containing 30 mM Bis-Tris (pH 6.2), 50 mM NaCl, 10 mM MgCl_2_, 0.5 mM (10 nmol) [γ^32^P]ATP, 1 mM (20 nmol) IP_6_, and Asp1-(1-385) as specified were incubated at 37°C for 90 min. The extents of IP_7_ and P_i_ formation are plotted as a function of input enzyme. (E) Kinetic profile. A reaction mixture (90 μL) containing 30 mM Bis-Tris (pH 6.2), 50 mM NaCl, 10 mM MgCl_2_, 0.5 mM [γ^32^P]ATP, 1 mM IP_6_, and 5 μM Asp1-(1-385) was incubated at 37°C. At times specified, aliquots (10 μL; containing 5 nmol ATP, 10 nmol IP_6_, and 50 pmol enzyme) were withdrawn and quenched immediately by adjustment to 45 mM EDTA. The extents of IP_7_ and P_i_ formation are plotted as a function of time. (F) Divalent cation specificity for ATP hydrolysis. Reaction mixtures (20 μL) containing 30 mM Tris-acetate (pH 6.0), 50 mM NaCl, 0.5 mM (10 nmol) [γ^32^P]ATP, 5 μM (100 pmol) Asp1-(1-385), and either no metal (–) or 2 mM the indicated divalent cation (as the chloride salt, except for CdSO_4_) were incubated for 90 min at 37°C. The extents of P_i_ formation are plotted in bar graph format. All data in the graphs in panels A–F are the averages of three independent experiments ± SEM.

IP_6_ kinase activity required exogenous magnesium; ATP hydrolysis (a low level of which was detectable in the absence of magnesium) was strongly stimulated by inclusion of magnesium (Fig. 1B, right panel, and Fig. 2A). Whereas ATP hydrolysis in the presence of IP_6_ was optimal at 0.5 to 1 mM magnesium, IP_6_ kinase activity required higher magnesium concentrations (Fig. 2A). IP_7_ formation increased linearly with Mg^2+^ up to 2 mM and was optimal at 5 to 10 mM (Fig. 2A). The reasons for the higher Mg^2+^ requirement for IPP kinase activity versus ATPase can be surmised from the crystal structure of PPIP5K2 captured as a transition-state mimetic with ADP•MgF_3_ and 5-IP_7_ in the active site (14). This structure revealed two catalytic magnesium ions that engage the three ATP phosphates and stabilize the pentacoordinate phosphorane transition state of the γ-phosphate. The structure also disclosed two additional magnesium ions that are engaged to the IP_7_ phosphate groups not directly involved in kinase reaction chemistry. One Mg^2+^ forms a hexa-hydrated complex that makes water-mediated contacts to the 2 and 3 phosphates of IP_7_. Another Mg^2+^ forms a tetra-hydrated coordination complex that makes direct and water-mediated contacts to the 4 phosphate and 5 pyrophosphate groups of IP_7_. These two noncatalytic Mg^2+^ complexes also make water-mediated contacts to the enzyme. We infer that the noncatalytic magnesium ions are important for productive binding of the inositol polyphosphate substrate to the kinase active site but may be irrelevant to the ATPase reaction.

The effect of varying pH on the reaction of Asp-(1-385) with [γ^32^P]ATP and IP_6_ is shown in Fig. 2B. In this experiment, the Mg^2+^ concentration was adjusted to 2 mM to avoid the formation of an insoluble Mg^2+^-IP_6_ precipitate that occurred at pH ≥7.5 when the Mg^2+^ concentration was 5 mM or greater. IP_6_ kinase activity displayed a bell-shaped pH curve with optimal activity between pH 5.5 and pH 7.0 in Tris buffer. ATP hydrolysis in the presence of IP_6_ was optimal at pH 5.5 to 6.0 and plateaued between pH 6.5 and pH 9.5 on the alkaline side, while sharply falling off on the acidic side of the activity peak (Fig. 2B). ATPase activity in the absence of IP_6_ displayed a sharper pH optimum peaking at pH 5.5 to 6.0 and tailing off steadily at higher pH values (Fig. 2C).

The extents of IP_7_ and P_i_ product formation in IP_6_-containing reactions with 10 mM Mg^2+^ at pH 6.2 increased in lockstep with the amount of Asp1-(1-385) protein added (Fig. 2D). At limiting enzyme, 5 pmol of Asp1-(1-385) generated 490 pmol of IP_7_ and 390 pmol of P_i_ in 90 min, which translates into turnover numbers of ~1.1 min^−1^ and ~0.86 min^−1^ for kinase and ATPase activities, respectively. The kinetic profile of product formation by 5 μM Asp1-(1-385) affirmed that IP_7_ and P_i_ accumulated in tandem (Fig. 2E). Taking the 5 min time point as indicative of initial rate, we calculated turnover numbers of 1.14 min^−1^ and 0.64 min^−1^ for the kinase and ATPase activities, respectively. At the 120 min time point in this experiment, 54% of the input [γ^32^P]ATP substrate had been consumed and converted to IP_7_ plus P_i_ products. Our apparent turnover number of 1.14 min^−1^ for the IP_6_ kinase activity of the isolated kinase domain of fission yeast Asp1 is in the same range as the *k*_cat_ value of 0.6 min^−1^ reported for IP_6_ kinase activity of full length Asp1 (10) and the *k*_cat_ of 1.8 min^−1^ reported for IP_6_ phosphorylation by the kinase domain of human PPIP5K2 (13).

To gauge the metal cofactor specificity of Asp1-(1-385), we tested various divalent cations at 2 mM concentration for their ability to support ATP hydrolysis at pH 6.0 in the absence of IP_6_. (IP_6_ was omitted in light of its propensity to form an insoluble precipitate in the presence of several of the transition metals that we planned to test.) Magnesium, manganese, and cobalt ions were equally adept at supporting ATPase activity (Fig. 2F). Other metal ions were less effective in descending order as follows: nickel, cadmium, calcium, copper, zinc (Fig. 2F).

### NTP donor specificity of Asp1 kinase.

**FIG 3:**
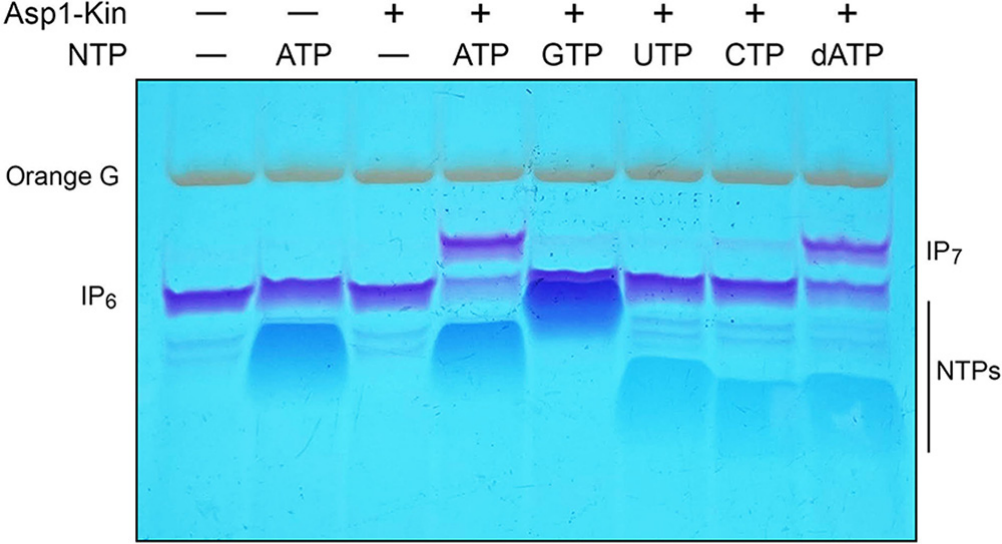
NTP donor specificity of Asp1 kinase. Reaction mixtures (20 μL) containing 30 mM Bis-Tris (pH 6.2), 50 mM NaCl, 10 mM MgCl_2_, 1 mM IP_6_, 5 μM Asp1-(1-385) (lanes +), and either no added NTP (–) or 5 mM ATP, GTP, UTP, CTP, or dATP as specified were incubated at 37°C for 90 min. Reaction products were analyzed by PAGE and detected by toluidine blue staining.

To query the NTP requirement for the kinase reaction, we implemented an assay in which Asp1 was incubated for 90 min with 1 mM IP_6_, 10 mM MgCl_2_, and 5 mM cold nucleoside triphosphate (either ATP, GTP, UTP, CTP, or dATP). The reactions were quenched with EDTA and the products were analyzed by electrophoresis through a 36% polyacrylamide gel. The polyphosphorylated species were visualized by staining the gel with toluidine blue (20). Conversion of input IP_6_ substrate to a slower-migrating IP_7_ product depended on inclusion of both ATP (which migrated ahead of IP_6_) and Asp1 kinase enzyme (Fig. 3). Whereas Asp1 kinase appeared equally adept at using 5 mM ATP and 5 mM dATP as phosphate donors, there was only scant formation of IP_7_ in the presence of 5 mM GTP or CTP and no IP_7_ generated in the presence of 5 mM UTP. The adenine nucleobase specificity of Asp1 kinase is consistent with the reported structure of the human PPIP5K2 homolog in complex with ATP, which highlights atomic contacts of conserved glutamate and lysine side chains with the adenine N6 and N7 atoms, respectively (see Fig. 5B). Whereas a conserved aspartate makes bidentate hydrogen bonds to the adenosine 2′-OH and 3′-OH groups, our results anent dATP suggest that the 2′-OH interaction is not critical under the assay conditions employed (i.e., at high NTP concentration).

### Asp1 kinase phosphorylates 5-IP_7_ but not 1-IP_7_.

**FIG 4:**
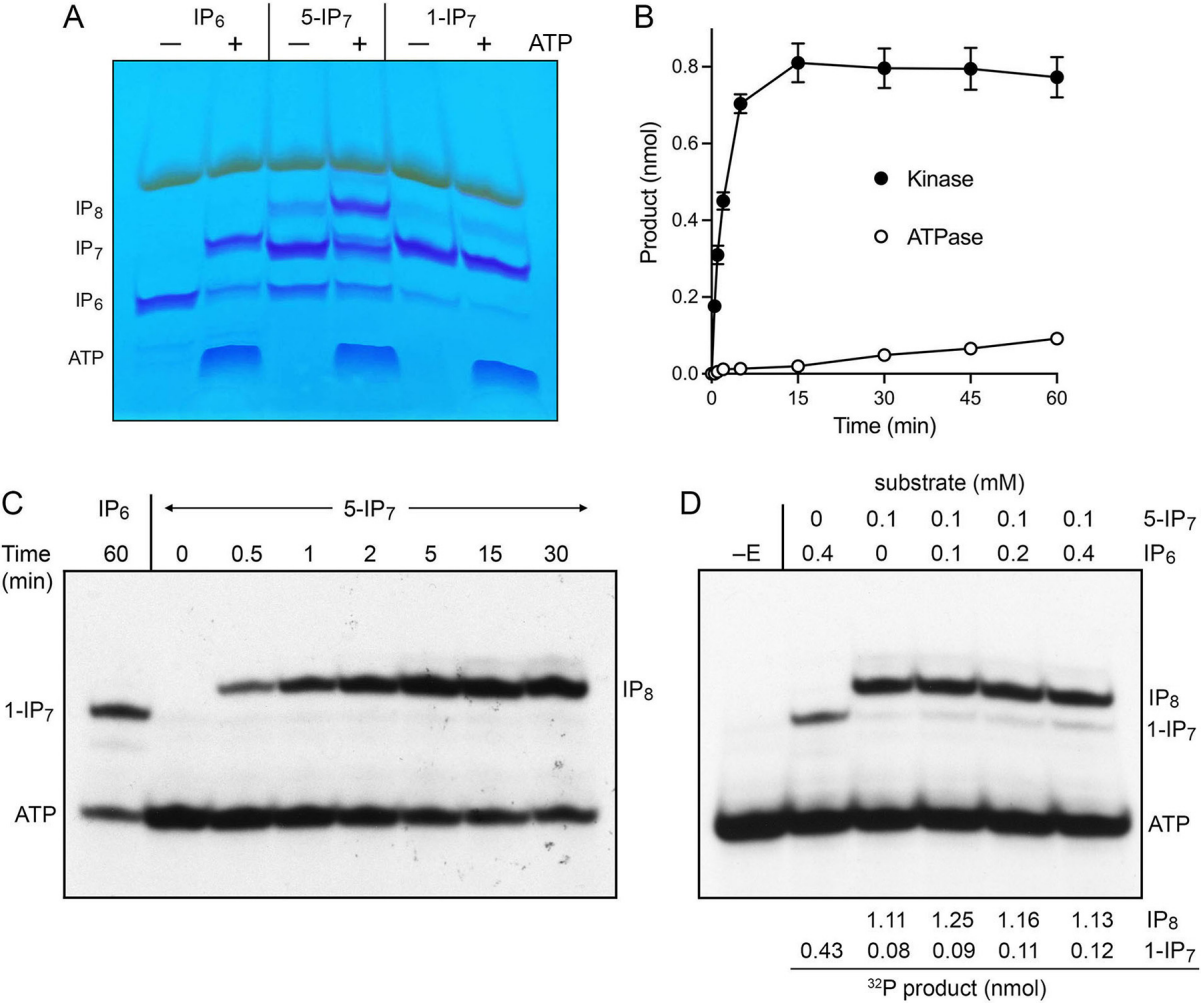
5-IP_7_ kinase activity. (A) Reaction mixtures (20 μL) containing 30 mM Bis-Tris (pH 6.2), 50 mM NaCl, 5 mM MgCl_2_, 2 mM ATP (in lanes labeled +), 0.5 mM IP_6_, 5-IP_7_, or 1-IP_7_ as specified, and 5 μM Asp1-(1-385) were incubated at 37°C for 90 min. Reaction products were analyzed by PAGE and detected by toluidine blue staining. (B) Kinetic profile. A reaction mixture (50 μL) containing 30 mM Bis-Tris (pH 6.2), 50 mM NaCl, 5 mM MgCl_2_, 0.25 mM [γ^32^P]ATP, 0.5 mM 5-IP_7_, and 2.5 μM Asp1-(1-385) was incubated at 37°C. At times specified, aliquots (5 μL; containing 1.25 nmol ATP, 2.5 nmol input 5-IP_7_, and 12.5 pmol enzyme) were withdrawn and quenched immediately with EDTA. The products were analyzed by TLC. The extents of IP_8_ formation (kinase) and P_i_ formation (ATPase) are plotted as a function of time. The data are averages of three independent experiments ± SEM. (C) A 5-IP_7_ kinase reaction was performed as in panel B. Aliquots quenched at the times specified were analyzed by PAGE and the ^32^P-labeled species were visualized by autoradiography. A control Asp1 kinase reaction with IP_6_ as substrate was incubated for 60 min and analyzed in parallel. The positions of ^32^P-labeled ATP substrate and the 1-IP_7_ and IP_8_ kinase products are indicated at left and right. (^32^Pi runs off the bottom of the gel and is not visualized.) (D) Reaction mixtures (20 μL) containing 30 mM Bis-Tris (pH 6.2), 50 mM NaCl, 5 mM MgCl_2_, 0.25 mM (5 nmol) [γ^32^P]ATP, IP_6_ and 5-IP_7_ at the concentrations specified, and 2.5 μM (50 pmol) Asp1 kinase were incubated at 37°C for 15 min. Asp1 kinase was omitted from a control reaction (lane –E). Products were analyzed by PAGE; an autoradiograph of the gel is shown. The gel was scanned with a Typhoon FLA7000 imager and radioactivity was quantified with ImageQuant-TL. Yields of ^32^P-labeled IP_8_ and 1-IP_7_ were calculated by normalizing their signal intensities to that of ATP in the no enzyme control.

Asp1 kinase was reacted for 90 min with 2 mM ATP, 5 mM MgCl_2_, and either 0.5 mM chemically synthesized 5-IP_7_ or 1-IP_7_ (21–23) or 0.5 mM IP_6_. The Asp1 kinase products were analyzed by 36% PAGE (lanes + in Fig. 4A), in parallel with control samples containing the inositol polyphosphate substrate but lacking ATP and kinase (lanes –). Whereas Asp1 kinase catalyzed the conversion of 5-IP_7_ into a more slowly migrating IP_8_ product, no such conversion was observed when the kinase was reacted with 1-IP_7_ (Fig. 4A). These results affirm previous reports that Vip1/PPIP5K kinase enzymes are specific for phosphorylation at the 1-phosphate position (10, 14). (Note: the preparations of 5-IP_7_ and 1-IP_7_ contained additional toluidine blue-staining species as minor impurities, one of which is residual IP_6_ [with which it comigrated during PAGE] and another, migrating slower than IP_7_, that corresponds to an IP_7_ species that was incompletely deprotected).

To quantify Asp1 activity with 5-IP_7_ as substrate, we tracked the kinetics of product formation in a reaction containing 0.5 mM 5-IP_7_, 0.25 mM [γ^32^P]ATP, 5 mM MgCl_2_, and 2.5 μM Asp1 kinase. The products were analyzed by TLC and the extents of ATP hydrolysis to ^32^P_i_ and of label transfer from ATP to form ^32^P-IP_8_ were plotted as a function of reaction time (Fig. 4B). Unlike the IP_6_ kinetic profile documented in Fig. 2E, in which the reaction partitioned nearly equally between kinase and ATPase outcomes, the kinetic profile with 5-IP_7_ was strongly biased in favor of the kinase reaction. At the 15 min time point, 65% of the input ATP was consumed in the kinase reaction versus 2% in the ATPase reaction (Fig. 4B). From the initial rate of the IP_7_ kinase reaction, we calculated a turnover number of 25.4 min^−1^, a value 22-fold greater than the apparent rate of the IP_6_ kinase reaction in Fig. 2E.

A separate analysis of the kinetic profile of the IP_7_ kinase reaction was performed by subjecting the ^32^P-labeled products to PAGE and visualization by autoradiography (Fig. 4C). This experiment showed clearly that the radiolabeled product of the IP_7_ kinase reaction was IP_8_, which migrated more slowly than the 1-IP_7_ produced in a parallel reaction with IP_6_ as the substrate (Fig. 4C).

Metabolic labeling with ^3^H-inositol had revealed that the intracellular concentration of IP_6_ in fission yeast is ~10-fold higher than that of 5-IP_7_ (9). Therefore, it was of interest to gauge the product distribution of the Asp1 kinase reaction when the enzyme was presented with 0.25 mM [γ^32^P]ATP and a mixture of 5-IP_7_ and IP_6_ as phosphate acceptors, versus 5-IP_7_ or IP_6_ alone. The yield of ^32^P-IP_8_ in a reaction containing 0.1 mM 5-IP_7_ alone was 2.6-fold higher than the yield of ^32^P-IP_7_ in a reaction containing 0.4 mM IP_6_ alone (Fig. 4D). The salient findings were that inclusion of 0.1, 0.2, or 0.4 mM IP_6_ in reactions containing 0.1 mM 5-IP_7_ had scant effect on the yield of ^32^P-IP_8_. To wit, 9.4-fold more IP_8_ than 1-IP_7_ was produced even when IP_6_ was present in 4-fold excess over 5-IP_7_. Thus, under competitive conditions, 5-IP_7_ is the preferred kinase substrate and 1,5-IP_8_ synthesis is the preferred reaction outcome.

### Structure-function analysis by alanine scanning.

**FIG 5:**
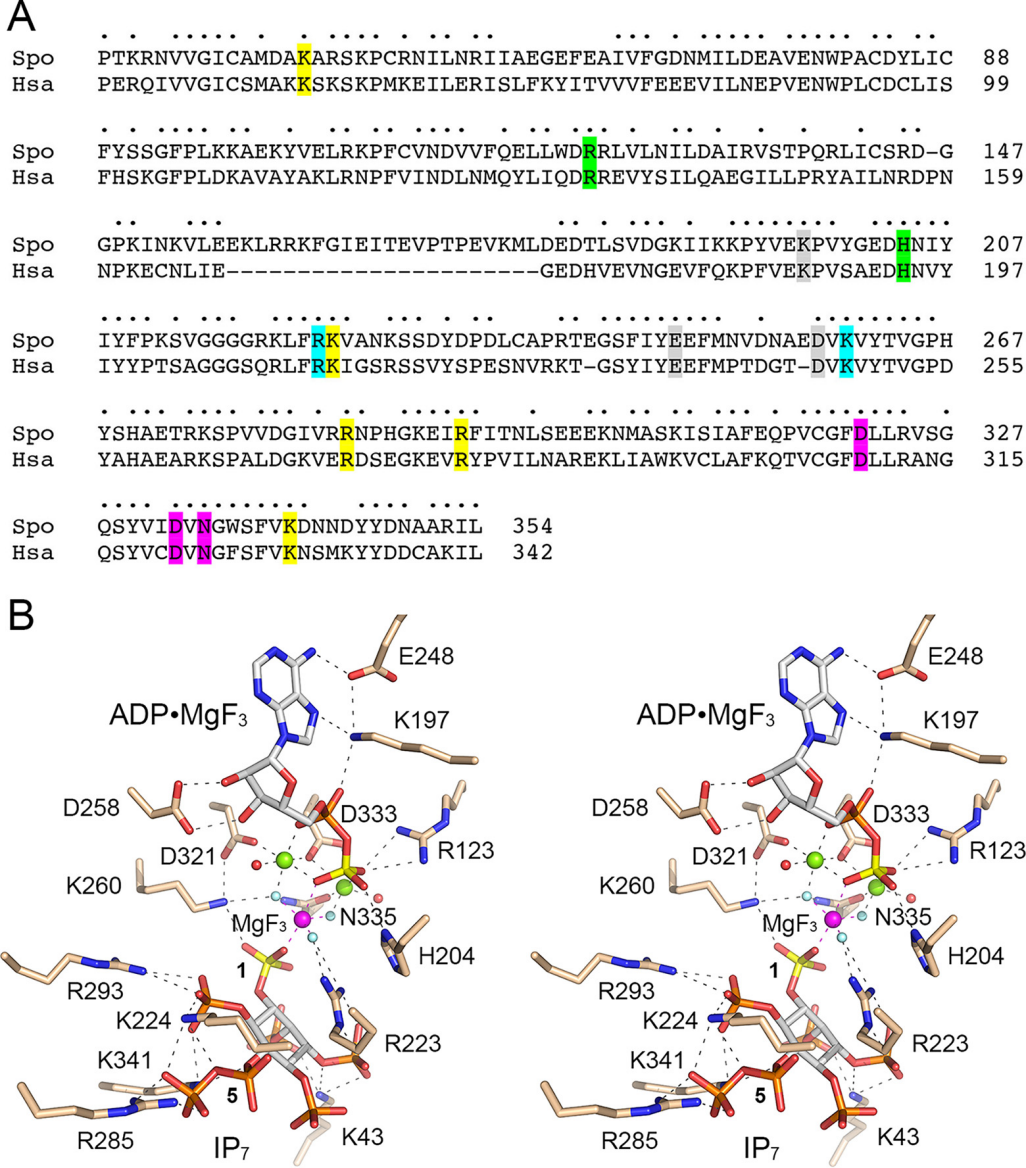
Structural homology of fission yeast Asp1 and human PPIP5K2 kinase domains. (A) The amino acid sequence of the S. pombe (Spo) Asp1 kinase domain is aligned to that of the Homo sapiens (Hsa) PPIP5K2 kinase domain. Positions of amino acid side chain identity/similarity are denoted by dots above the alignment. Gaps in the alignment are indicated by dashes. Amino acids in PPIP5K2 are highlighted in colored shading according to their contacts in the active site structure depicted in panel B. PPIPK2 residues that coordinate the two magnesium cofactors are highlighted in magenta. Residues that engage the adenosine nucleoside and the ADP β-phosphate are shaded in gray and green, respectively. Residues that coordinate the MgF_3_ mimetic of the γ-phosphate transition state or the 1-phosphate of 5-IP_7_ are shaded in cyan. Residues that contact the other phosphates of 5-IP_7_ are highlighted in yellow. (B) Stereo view of the active site of PPIPK2 (from PDB 3T9E) as the ADP•MgF_3_ transition state mimetic (MgF_3_ rendered as atomic spheres with magnesium colored magenta and fluorines colored pale blue) in complex with 5-IP_7_ (stick model with gray carbons) and two magnesium cofactors (green spheres). The ADP β-phosphorus and 5-IP_7_ 1-phosphorus atoms are colored yellow. Waters are depicted as red spheres. Amino acids are rendered as stick models with beige carbons. Amino acids are numbered according to their conserved counterparts in Asp1.

**FIG 6:**
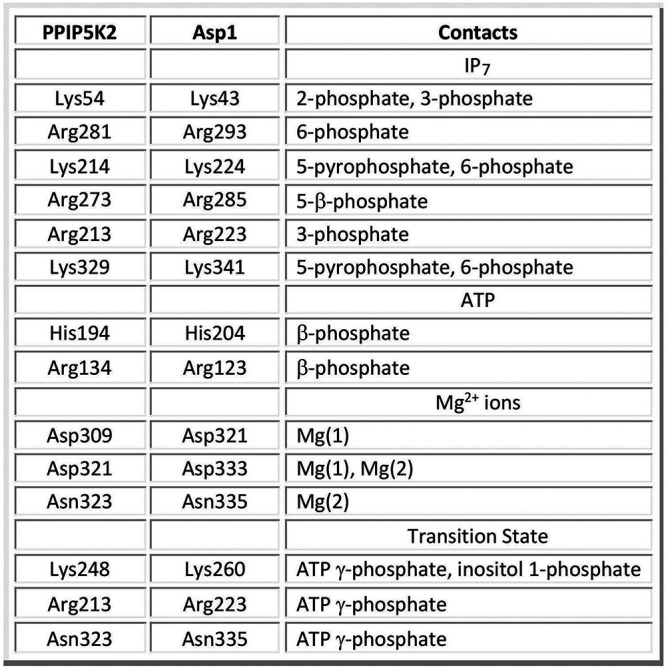
Amino acids targeted for mutagenesis in Asp1 kinase and their conserved counterparts in human PPIP5K2.

**FIG 7:**
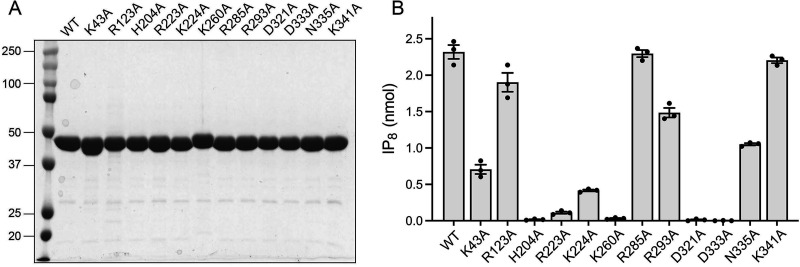
Structure-guided alanine scanning mutagenesis of Asp1 kinase. (A) Aliquots (8 μg) of wild-type Asp1 kinase domain and the indicated alanine mutants were analyzed by SDS–PAGE. The Coomassie-blue stained gel is shown. The positions and sizes (kDa) of marker polypeptides (leftmost lane) are indicated. (B) Kinase reaction mixtures (20 μL) containing 30 mM Bis-Tris (pH 6.2), 50 mM NaCl, 5 mM MgCl_2_, 0.25 mM (5 nmol) [γ^32^P]ATP, 0.5 mM (10 nmol) 5-IP_7_, and 2.5 μM (50 pmol) of wild-type Asp1 kinase domain or the indicated alanine mutants were incubated at 37°C for 15 min. The products were analyzed by TLC. The extents of IP_8_ formation are plotted for each enzyme. The data in the bar graph are the averages of three independent experiments ± SEM.

We used a primary structure alignment of the human PPIP5K2 and fission yeast Asp1 kinase domains (Fig. 5A) and the atomic structure of a PPIP5K2 kinase transition state mimetic (Fig. 5B) (14) to guide an alanine scan of the Asp1 kinase active site. The human and fission yeast kinases share 197 positions of amino acid side chain identity/similarity over the segment of Asp1 spanning aa 29 to 342 (Fig. 5A). A stereo view of the transition state structure of PPIP5K2 is shown in Fig. 5B with amino acid side chains numbered according to their identical counterparts in Asp1. The conserved active site residues that contact the ATP adenosine, the β-phosphate, and the MgF_3_ mimetic of the γ-phosphate transition state are shaded gray, green, and cyan, respectively, in Fig. 5A. The amino acids that coordinate the catalytic magnesium ions and the IP_7_ phosphates are shaded magenta and yellow, respectively in Fig. 5A. Here we introduced alanine in lieu of 12 Asp1-(1-385) amino acids that are predicted to engage the IP_7_ phosphate acceptor, the ATP phosphate donor, and the catalytic magnesium ions. The Asp1 amino acids targeted, their counterparts in the human PPIP5K2, and their atomic contacts are compiled in Fig. 6. The recombinant Ala-mutants were produced in E. coli in parallel with the wild-type Asp1-(1-385). SDS-PAGE analysis of the respective peak Superdex-200 fractions is shown in Fig. 7A.

Equivalent amounts of wild-type and mutant proteins were assayed for activity with 5-IP_7_ substrate (Fig. 7B). Three classes of mutational effects were observed: (i) those that eliminated IP_7_ kinase activity or reduced product formation to less than 5% of wild type (H204A, R223A, K260A, D321A, and D333A); (ii) those that did not affect IP_7_ kinase activity (R285A and K341A); and (iii) those that had displayed modestly reduced activity *vis-à-vis* wild type: R123A (82% of WT), R293A (64%), N335A (45%), K43A (30%), and K224A (18%) (Fig. 7B).

The essential Asp321 and Asp333 side chains are the enzymatic ligands for the two catalytic metal ions. The essential His204 coordinates the ATP β-phosphate in the human PPIP5K2 structure. The essential Lys260 coordinates the ATP γ-phosphate and the inositol 1-phosphate. The essential Arg223 coordinates the ATP γ-phosphate and the inositol 3-phosphate. It is noteworthy that Asn335, which in the human PPIP5K2 structure coordinates the Mg(2) metal cofactor and the ATP γ-phosphate, is apparently not essential for catalysis by Asp1. Neither is Arg123, which contacts the ATP β-phosphate.

Other mutations that spare or moderately diminish Asp1 IP_7_ kinase activity are mainly those that subtract predicted side chain contacts to the 5-IP_7_ substrate remote from the 1-phosphate site at which chemistry occurs: Arg285, predicted to engage the 5-β-phosphate; Lys341, the 5-β-phosphate and 6-phosphate; Arg293, the 6-phosphate; Lys43, the 2- and 3-phosphates; and Lys224, the 5-β-phosphate and 6-phosphate. We suspect there is functional redundancy among the Asp1 amino acids that make atomic contacts to the same phosphate groups of the 5-IP_7_ substrate. Also, the effects of subtracting a single remote phosphate contact on kinase activity (via a putative effect on affinity for 5-IP_7_) might well be obscured by our assay conditions wherein the 5-IP_7_ concentration (0.5 mM) greatly exceeds the reported *K*_m_ of 0.06 μM for human PPIP5K2 (13).

### Asp1 kinase activity de-represses *pho1* expression *in vivo*.

Transcriptome profiling of IPP pyrophosphatase-defective *asp1-H397A* cells has delineated an IPP-responsive regulon comprising 30 protein-coding genes that were overexpressed when cellular IP_8_ levels are increased (19). The “top hits” with respect to fold upregulation included the phosphate-regulated genes: *tgp1* (up 21-fold) and *pho1* (up 7-fold). Transcriptome profiling of the IPP-kinase defective *asp1-D333A* strain highlighted that phosphate homeostasis genes *pho1* and *pho84* were downregulated, by 20-fold and 14-fold, respectively, in the absence of cellular IP_8_. Quantitative assay of cell surface-associated Pho1 acid phosphatase activity provides a convenient gauge of *pho1* gene expression under steady-state conditions, which recapitulates the derepression and hyper-repression of Pho1 activity in *asp1-H397A* and *asp1-D333A* mutants vis-à-vis wild-type cells (19). To gauge the impact of Asp1 kinase mutations *in vivo*, we established a Pho1 activity-based reporter system in which pTIN plasmids expressing wild-type or mutated versions of Asp1-(1-385) were introduced into *asp1*Δ cells. The pTIN expression vector (24) places the Asp1-(1-385) open reading frame under the transcriptional control of the *tgp1* promoter, which is situated adjacent to the transcription unit specifying the *nc-tgp1* lncRNA driven by the thiamine-repressible *nmt1* promoter. In the absence of thiamine, lncRNA transcription interferes with firing of the *tgp1* promoter. In the presence of thiamine, lncRNA synthesis is turned off and expression of the downstream mRNA—encoding Asp1-(1-385) in this case—is turned on (24).

**FIG 8:**
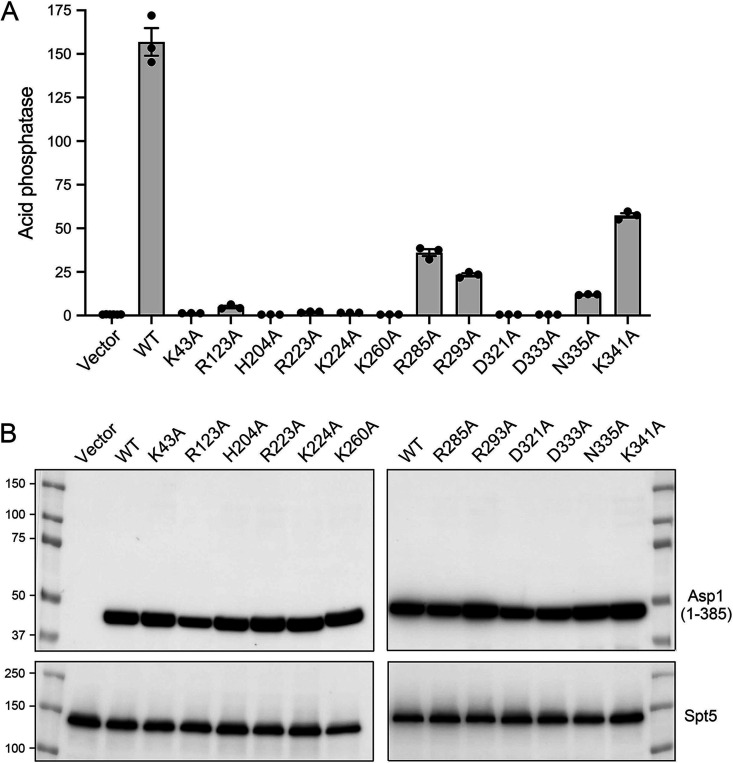
Asp1 kinase activity derepresses *pho1* expression *in vivo*. (A) Single colonies (≥20) of *asp1*Δ cells bearing either pTIN plasmids encoding wild-type or mutant Asp1 kinase domains or the empty pTIN vector were pooled and grown in Leu^−^ ePMG with 15 μM thiamine. Aliquots of exponentially growing cultures were assayed for acid phosphatase activity. The data are averages (±SEM) of at least three independent biological replicates. (B) Western blots of whole-cell extracts prepared from *asp1*Δ cells bearing the indicated pTIN plasmids. The blots were probed with affinity-purified rabbit polyclonal antibodies recognizing Asp1 or Spt5 (as a loading control), as specified. The positions and sizes (kDa) of protein markers are indicated on the left.

The dynamic range of the assay was established by comparing Pho1 acid phosphatase activity of thiamine-replete *asp1*Δ cells bearing the pTIN-Asp1-(1-385) wild-type plasmid versus cells bearing the empty pTIN vector. Acid phosphatase activity was quantified by incubating suspensions of serial dilutions of the cells for 5 min with *p*-nitrophenylphosphate and assaying colorimetrically the formation of *p*-nitrophenol. Activity is expressed as the ratio of *A*_410_ (*p*-nitrophenol production) to *A*_600_ (input cells). *asp1*Δ cells with the empty pTIN vector had a Pho1 activity level of 0.6, whereas cells expressing the wild-type Asp1 kinase domain from the pTIN vector had an activity level of 157 (Fig. 8A). As a reference point, *asp1*^+^ cells have a Pho1 activity level of 4.5 when grown in the same thiamine-replete ePMG liquid medium. Thus, expressing the isolated IPP kinase domain of Asp1 strongly de-repressed expression of the *pho1* gene from its native chromosomal locus.

### Effect of Asp1 kinase domain mutations on *pho1* expression.

Western blotting of whole-cell extracts of *asp1*Δ fission yeast bearing wild-type or mutant pTIN-Asp1-(1-385) plasmids using affinity-purified anti-Asp1 antibody revealed that the steady-state levels of Asp1 kinase protein were similar in *asp1*Δ strains expressing wild-type and mutant Asp1 kinases, with the exception of R123A (Fig. 8B). As expected, there was no Asp1 kinase protein detected in *asp1*Δ cells bearing the empty pTIN vector (Fig. 8B). Assays of cell-surface acid phosphatase activity revealed that mutations H204A, R223A, K260A, D321A, and D333A, which abolished or nearly abolished IPP kinase activity, effaced the derepression of Pho1 when mutant proteins were expressed from pTIN plasmids (Fig. 8A). These results affirm that the IPP kinase function of Asp1 is what drives Pho1 de-repression *in vivo*.

Among the mutations that retained partial IP_7_ kinase activity *in vitro*, K224A and K43A (with the lowest kinase activities) were unable to derepress Pho1 *in vivo*, whereas R285A, R293A, K341A, and N335A did derepress Pho1 (by 62-fold, 40-fold, 98-fold, and 21-fold, respectively compared to the vector control), albeit not to the degree achieved by wild-type Asp1 kinase (Fig. 8A). R123A retained partial kinase activity *in vitro* but derepressed Pho1 by only 8-fold when expressed with the pTIN system (Fig. 8A), an effect we would attribute to the apparently lower steady-state level of the R123A kinase polypeptide *vis-à-vis* wild-type and the other mutants (Fig. 8B).

### Characterization of recombinant full-length Asp1.

**FIG 9:**
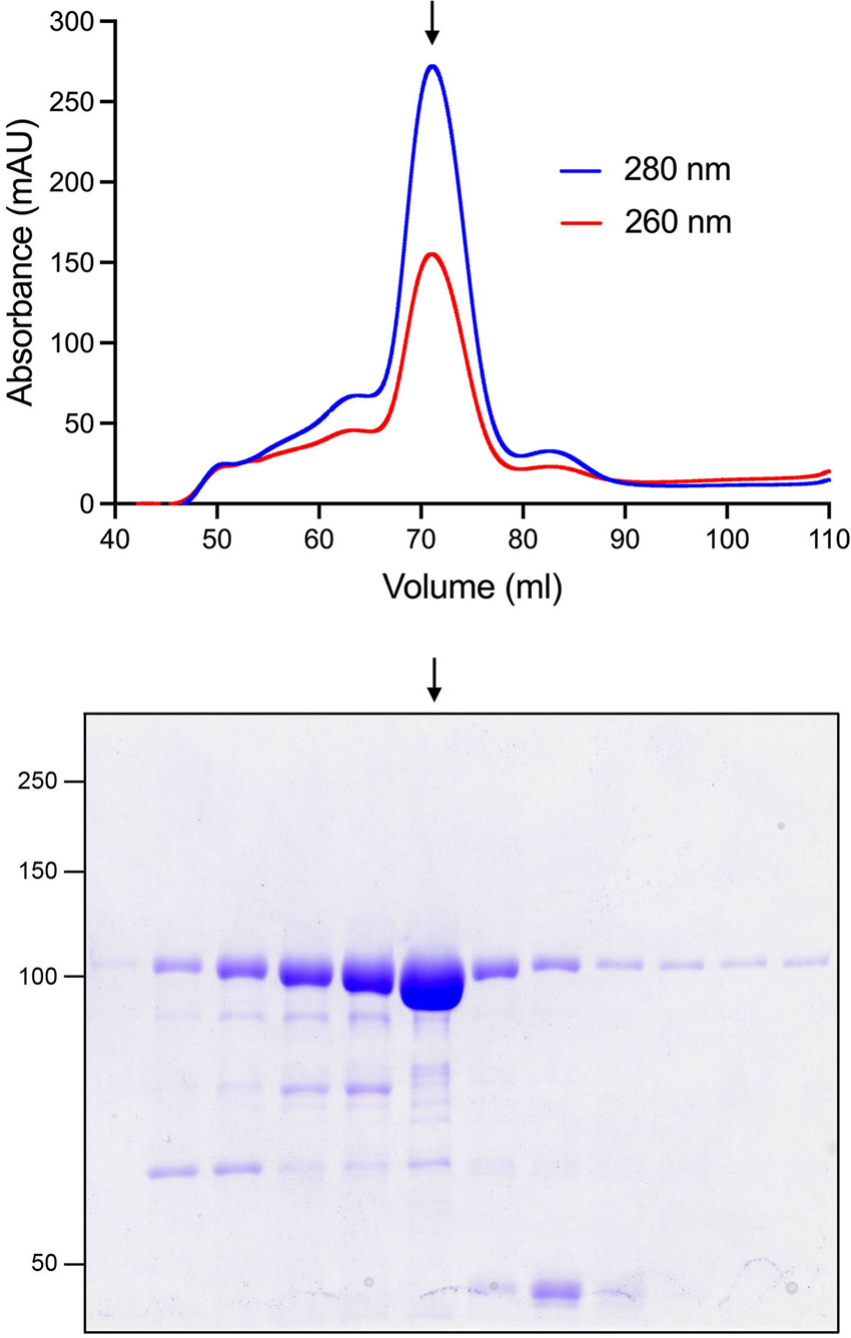
Recombinant full-length Asp1. Elution profile of wild-type Asp1 during Superdex-200 gel filtration. The top panel shows the absorbance at 280 nm (blue trace) and 260 nm (red trace) as a function of elution volume. The *A*_260_=*A*_280_ peak at 50 mL demarcates the void volume. Aliquots (5 μL) of the fractions spanning and flanking the *A*_280_ peak were analyzed by SDS-PAGE. The Coomassie blue-stained gel is shown. The positions and sizes (kDa) of marker proteins are indicated on the left. The fraction corresponding to the *A*_280_ peak containing purified Asp1 is indicated by the vertical arrow.

**FIG 10:**
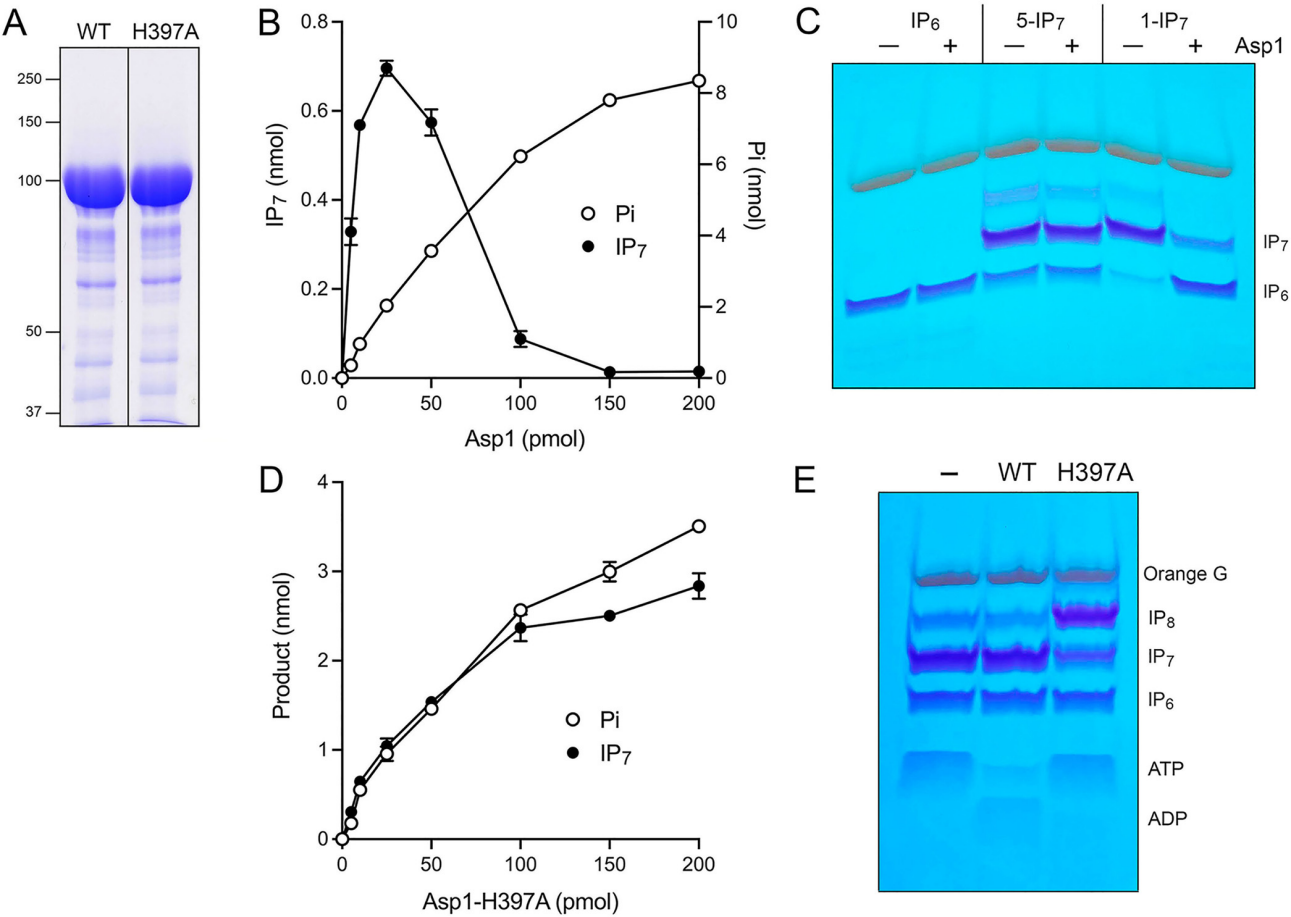
Biochemical activities of full-length Asp1 and active site mutant. (A) Aliquots (15 μg) of full-length wild-type Asp1 and H397A mutant were analyzed by SDS–PAGE. The Coomassie-blue stained gel is shown. The positions and sizes (kDa) of marker polypeptides are indicated on the left. (B) Reaction mixtures (20 μL) containing 30 mM Bis-Tris (pH 6.2), 50 mM NaCl, 10 mM MgCl_2_, 0.5 mM (10 nmol) [γ^32^P]ATP, 1 mM IP_6_, and Asp1 as specified were incubated at 37°C for 90 min. Products were analyzed by TLC. The extents of IP_7_ and P_i_ formation are plotted as a function of input enzyme. (C) Phosphatase reaction mixtures (20 μL) containing 30 mM Bis-Tris (pH 6.2), 50 mM NaCl, 2 mM MgCl_2_, 0.5 mM IP_6_, 5-IP_7_ or 1-IP_7_, and 5 μM wild-type Asp1 (lanes +) were incubated at 37°C for 30 min. Reaction products were analyzed by PAGE and detected by toluidine blue staining. (D) Kinase reaction mixtures (20 μL) containing 30 mM Bis-Tris (pH 6.2), 50 mM NaCl, 10 mM MgCl_2_, 0.5 mM (10 nmol) [γ^32^P]ATP, 1 mM IP_6_, and Asp1-(H397A) as specified were incubated at 37°C for 90 min. The extents of IP_7_ and P_i_ formation are plotted as a function of input enzyme. The data in panels B and D are averages of three independent experiments ± SEM. (E) IP_7_ kinase reaction mixtures (20 μL) containing 30 mM Bis-Tris (pH 6.2), 50 mM NaCl, 5 mM MgCl_2_, 0.5 mM 5-IP_7_, 2 mM ATP, and 5 μM Asp1 WT or Asp1-(H397A) were incubated at 37°C for 30 min. Reaction products were analyzed by PAGE and detected by toluidine blue staining.

We produced full-length Asp1 in E. coli as a His_10_Smt3 fusion and purified the protein from a soluble bacterial extract by sequential Ni-agarose, tag cleavage, second Ni-agarose, and gel filtration steps. The elution profile of the 105 kDa Asp1 polypeptide was consistent with it being predominantly a monomer in solution (Fig. 9). In parallel, we purified a mutated version of Asp1 in which the pyrophosphatase active site residue His397 was changed to alanine. SDS-PAGE analysis of the wild-type and H397A full-length Asp1 preparations (peak gel filtration fractions) is shown in Fig. 10A.

Assay of the wild-type Asp1 for kinase activity with IP_6_ and [γ^32^P]ATP revealed a distinctly different enzyme titration profile (Fig. 10B) compared to that of the isolated kinase domain (Fig. 2D). Formation of ^32^P-IP_7_ peaked at 25 pmol of input Asp1, at which point 7% of the input ATP ^32^P-label was used in the kinase reaction (plotted on the left *y* axis scale in Fig. 10B) while 20% of the input ATP ^32^P-label was converted to ^32^P inorganic phosphate (plotted on the right *y* axis scale in Fig. 10B). Further increasing input Asp1 resulted in progressive reduction in the level of the kinase product (which was eliminated at ≥150 pmol of Asp1) as the extent of ^32^P phosphate formation steadily increased, to 82% of the input ATP ^32^P-label at 200 pmol Asp1 (Fig. 10B). We presume that the IPP pyrophosphatase activity resident in the C-terminal domain of Asp1 interferes with measurement of kinase activity because it effects the hydrolysis of the ^32^P-labeled 1-IP_7_ kinase reaction product.

To query the inositol pyrophosphatase activity of Asp1, we reacted the enzyme for 30 min with 0.5 mM IP_6_, 5-IP_7_, or 1-IP_7_ in the absence of ATP. Product analysis by PAGE showed that Asp1 converted 1-IP_7_ to IP_6_ but did not modify either the 5-IP_7_ or IP_6_ substrates (Fig. 10C), thereby affirming previous findings that Asp1 is specific for hydrolysis of the phosphoanhydride bond at the 1-pyrophosphate position (25).

To evade the complications of pyrophosphatase activity on kinase detection, we assayed the Asp1-H397A pyrophosphatase mutant for kinase activity with IP_6_ and [γ^32^P]ATP and found that it generated approximately equal distributions of kinase and ATPase reaction products, both of which increased steadily with input enzyme up to 200 pmol Asp1-H397A (Fig. 10D). PAGE-based assay of the wild-type and H397A full-length Asp1 proteins for 5-IP_7_ kinase activity in the presence of excess cold ATP is shown in Fig. 10E. Wild-type Asp1 failed to produce IP_8_ product and instead consumed most of the input ATP. (Note: ADP, the product of the kinase and ATPase reactions, is weakly stained by toluidine blue.) By contrast, Asp1-H397A did generate IP_8_ without significantly depleting the input ATP (Fig. 10E). We surmise that full-length wild-type Asp1 churns unproductively through futile cycles of IP_8_ synthesis by its kinase and decay by its pyrophosphatase.

### Effect of inorganic phosphate on activity of full-length Asp1.

**FIG 11:**
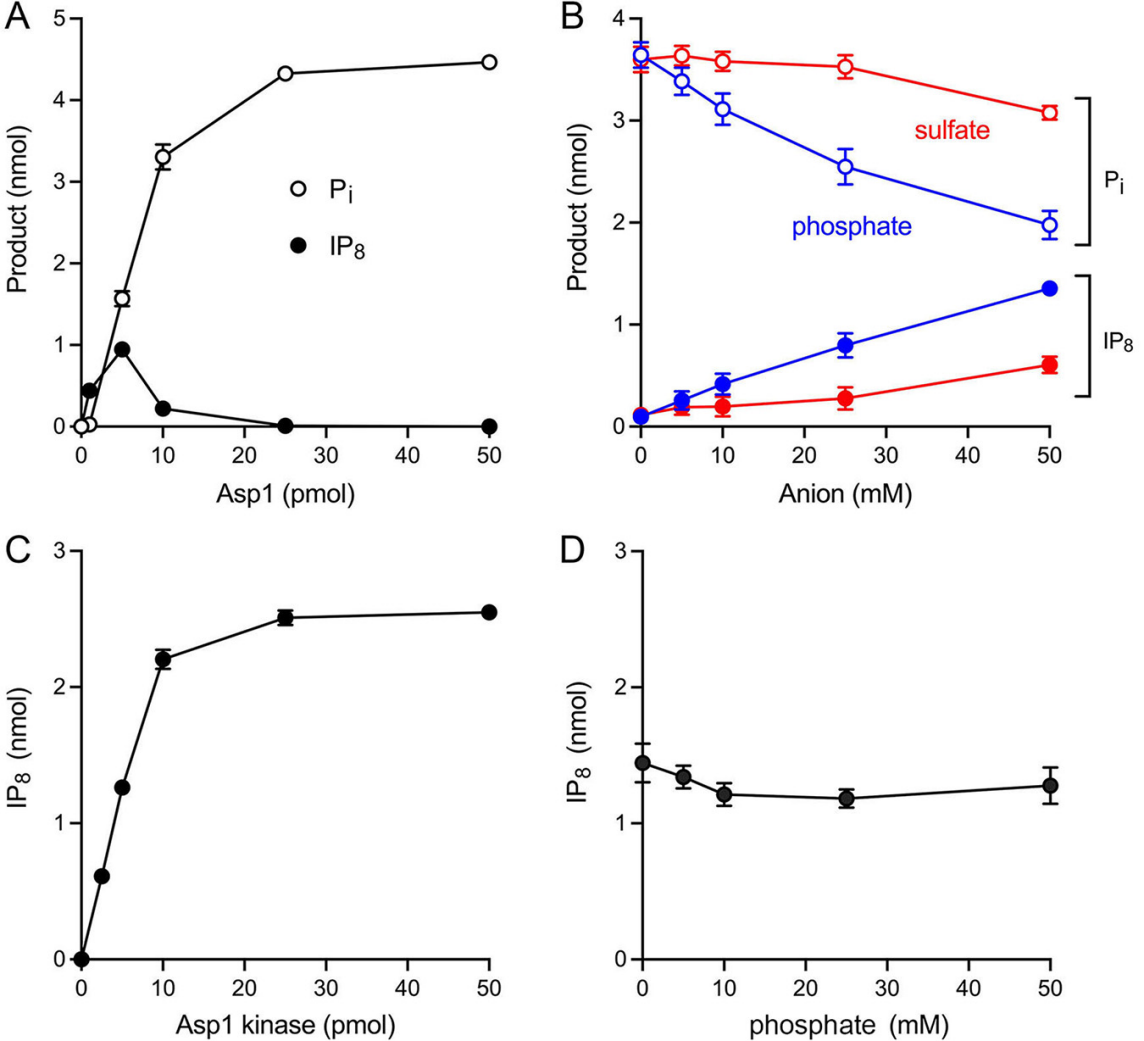
Effect of inorganic phosphate on activity of full-length Asp1. (A) Asp1 titration. Reaction mixtures (20 μL) containing 30 mM Bis-Tris (pH 6.2), 50 mM NaCl, 5 mM MgCl_2_, 0.25 mM (5 nmol) [γ^32^P]ATP, 0.5 mM 5-IP_7_, and full-length Asp1 as specified were incubated at 37°C for 30 min. Products were analyzed by TLC. The extents of ^32^P-IP_8_ and ^32^P_i_ formation are plotted as a function of input enzyme. (B) Effect of phosphate. Reaction mixtures (20 μL) containing 30 mM Bis-Tris (pH 6.2), 50 mM NaCl, 5 mM MgCl_2_, 0.25 mM (5 nmol) [γ^32^P]ATP, 0.5 mM 5-IP_7_, 10 pmol full-length Asp1, and sodium phosphate (pH 6.4) or sodium sulfate (pH 6.4) as specified were incubated at 37°C for 30 min. Products were analyzed by TLC. The extents of ^32^P-IP_8_ formation (closed circles) and ^32^P_i_ formation (open circles) are plotted as a function of phosphate or sulfate concentration. (C) Asp1 kinase titration. Reaction mixtures (20 μL) containing 30 mM Bis-Tris (pH 6.2), 50 mM NaCl, 5 mM MgCl_2_, 0.25 mM (5 nmol) [γ^32^P]ATP, 0.5 mM 5-IP_7_, and Asp1 kinase as specified were incubated at 37°C for 15 min. Products were analyzed by TLC. The extent of ^32^P-IP_8_ formation is plotted as a function of input enzyme. The data in panels A, B, and C are averages of three independent experiments ± SEM. (D) Effect of phosphate on Asp1 kinase domain. Reaction mixtures (20 μL) containing 30 mM Bis-Tris (pH 6.2), 50 mM NaCl, 5 mM MgCl_2_, 0.25 mM (5 nmol) [γ^32^P]ATP, 0.5 mM 5-IP_7_, 5 pmol Asp1 kinase domain, and sodium phosphate (pH 6.4) as specified were incubated at 37°C for 15 min. Products were analyzed by TLC. The extent of ^32^P-IP_8_ formation is plotted as a function of phosphate concentration. The data are the average of two independent experiments; error bars indicate the range of values.

Titration of full-length wild-type Asp1 for IP_7_ kinase activity via TLC assay showed that formation of ^32^P-IPP peaked at 5 pmol of input Asp1, at which point 19% of the input ATP ^32^P-label was present as IPP while 31% of the input ATP ^32^P-label was converted to ^32^P_i_ (Fig. 11A). Increasing Asp1 progressively reduced in the level of the kinase product (which was eliminated at ≥25 pmol of Asp1) as the extent of ^32^P phosphate formation steadily increased, to 89% of the input ATP ^32^P-label at 50 pmol Asp1 (Fig. 11A). These results, and those in Fig. 10, suggest that net IPP synthesis by full-length Asp1 is thwarted by the action of its IPP pyrophosphatase domain. Thus, for net IP_8_ synthesis by full-length Asp1 to be achieved *in vivo*, Asp1’s IPP pyrophosphatase activity must be susceptible to modulation. In this vein, the Shears lab has reported that the pyrophosphatase activity of human PPIP5Ks was inhibited by 5 mM inorganic phosphate and that net IP_8_ synthesis by PPIP5K2 was stimulated 2-fold by 5 mM P_i_ (26). By contrast, IP_8_ synthesis by the paralogous human IPP kinase/pyrophosphatase PPIP5K1 was insensitive to 5 mM P_i_ (26).

Here we tested the effect of increasing phosphate on net synthesis of IP_8_ by 10 pmol Asp1, an enzyme level at which product yield is biased toward ^32^P_i_ versus ^32^P-IPP. The instructive findings were that inorganic phosphate elicited a concentration-dependent shift in product formation in favor of net IPP synthesis (Fig. 11B). Compared to the no-phosphate control, supplementation with 5, 10, 25, and 50 mM phosphate increased the yields of IPP by factors of 2.7, 4.3, 8.3, and 14, respectively, concomitant with a concentration-dependent decrement in the formation of ^32^P_i_ (Fig. 11B). Sulfate was less effective than phosphate in stimulating net IPP synthesis at the expense of P_i_ formation (Fig. 11B); to wit, IPP yield on addition of 10, 25, and 50 mM sulfate was 1.7-fold, 2.4-fold, and 5.3-fold that of the unsupplemented control. We regard the 8-fold stimulation of net IP_8_ synthesis by 25 mM phosphate to be physiologically relevant insofar as the intracellular concentration of inorganic phosphate in budding yeast is 23 to 25 mM (as determined by ^31^P-NMR spectroscopy) (27).

To query the effect of phosphate on the IP_7_ kinase activity of the isolated Asp1 kinase domain, we first titrated the kinase domain in reactions containing 0.25 mM [γ^32^P]ATP and 0.5 mM 5-IP_7_ to establish a suitably sensitive enzyme concentration (Fig. 11C). The yield of ^32^P-IP_8_ product during a 15 min reaction increased with input Asp1 kinase; from the slope of the titration curve, we calculated that 228 ± 5 pmol of IP_8_ were formed per pmol of kinase. The effect of exogenous phosphate was assayed at 5 pmol of Asp1 kinase, a level at which half-maximal product formation was attained, thereby allowing for detection of either phosphate stimulation or inhibition of kinase activity. We found that inorganic phosphate had no impact on IP_8_ product formation at up to 50 mM concentration (Fig. 11D).

### Effect of full-length Asp1 on *pho1* expression.

**FIG 12:**
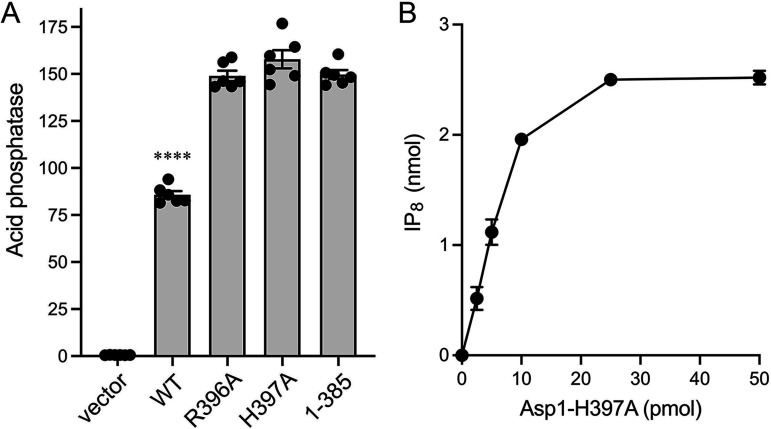
Effect of full-length Asp1 on *pho1* expression. (A) Single colonies (≥20) of *asp1*Δ cells bearing either the pTIN plasmids encoding full-length wild-type or mutant Asp1, the wild-type kinase domain (aa 1-385), or the empty pTIN vector were pooled and grown in Leu^−^ ePMG with 15 μM thiamine. Aliquots of exponentially growing cultures were assayed for acid phosphatase activity. The data are averages (± SEM) of six independent biological replicates. Whereas unpaired Welch’s *t* tests of the full-length Asp1 data versus that of the kinase domain showed no significant differences between kinase domain and Asp1-H397A (*P* value 0.173) or Asp1-R396A (*P* value 0.846), the full-length wild-type Asp1 differed significantly (*P* value < 0.0001, denoted by ****). (B) 5-IP_7_ kinase activity of Asp1-H397A. Reaction mixtures (20 μL) containing 30 mM Bis-Tris (pH 6.2), 50 mM NaCl, 5 mM MgCl_2_, 0.25 mM (5 nmol) [γ^32^P]ATP, 0.5 mM (10 nmol) 5-IP_7_, and Asp1-H397A as specified were incubated at 37°C for 15 min. Products were analyzed by TLC. The extent of ^32^P-IP_8_ formation is plotted as a function of input enzyme. The data are averages of three independent experiments ± SEM.

Expression of full-length wild-type Asp1 protein produced in *asp1*Δ cells via the pTIN system derepressed Pho1 activity, albeit only 57% as strongly as did the isolated kinase domain (Fig. 12A). This was to be expected given that the C-terminal IPP pyrophosphatase domain of Asp1 acts in opposition to the kinase domain and thus acts as a brake on the cellular accumulation of IP_8_. Pyrophosphatase domain mutations R396A and H397A of full-length Asp1, which eliminate pyrophosphatase activity *in vitro* (9), restored Pho1 derepression to the level achieved by the isolated kinase domain (Fig. 12A). This is in keeping with our finding that the 5-IP_7_ kinase titration profile of the full-length Asp1-H397A protein (Fig. 12B) indicated a specific activity of 202 ± 8 pmol of IP_8_ formed per pmol of enzyme, which was similar to the specific activity of the isolated kinase domain (Fig. 11C).

## DISCUSSION

The results herein fortify and extend our understanding of the enzymatic properties of the bifunctional Asp1 IPP kinase-pyrophosphatase of fission yeast, an ortholog of budding yeast Vip1 and human PPIP5K. Two lines of investigation were especially informative. First, our characterization of the autonomous Asp1 N-terminal kinase domain highlighted starkly different reaction rates and outcomes when the enzyme is presented with IP_6_ versus 5-IP_7_ as the phosphate acceptor substrate. Second, our analysis of full-length Asp1 revealed that (i) IP_8_ synthesis from 5-IP_7_ is effectively futile in the face of IP_8_ hydrolysis by the C-terminal pyrophosphatase component; and (ii) net synthesis of IP_8_ is enabled by physiological concentrations of inorganic phosphate that selectively antagonize IP_8_ turnover. As discussed below, our results have implications for IPP dynamics *in vivo* and the role of Asp1 in fission yeast phosphate homeostasis.

### Asp1 kinase.

We initiated biochemical studies of the recombinant kinase domain by utilizing commercially available IP_6_ as the phosphate acceptor substrate and an assay protocol that followed the fate of the ^32^P-labeled ATP γ-phosphate. We thereby discerned two reaction outcomes: formation of IP_7_ and ATP hydrolysis, which comprise productive and unproductive modes of catalysis, respectively, with respect to IPP metabolism. Whereas the human PPIP5K2 kinase domain had been shown to hydrolyze ATP unproductively in the presence of a phosphate acceptor (13, 14), this property had not been documented previously for Asp1 (9,10). Under reaction conditions optimal for IP_6_ kinase activity, we see that the Asp1 reaction path is split almost equally between kinase and ATPase and that ATP hydrolysis does not depend on IP_6_. By contrast, the ATPase of human PPIP5K2 kinase is strongly stimulated by the presence of IP_6_ and other inositol phosphates (13, 14, 28). By implementing an electrophoretic assay of IP phosphorylation in the presence of cold nucleoside triphosphates, we demonstrated that Asp1 kinase is specific for ATP and dATP as the phosphate donor and either inactive or feebly active with GTP, CTP, and UTP. The adenine requirement of Asp1 is consistent with the adenine nucleobase-specific enzymatic contacts seen in the human PPIP5K crystal structure.

Key insights emerged when we deployed synthetic 5-IP_7_ as the substrate for Asp1 kinase, in which case IP_8_ synthesis was favored by >30-fold over the hydrolysis of ATP. Indeed, the rate of phosphorylation of 5-IP_7_ by Asp1 kinase was 22-fold faster than the rate of IP_6_ phosphorylation. This result resonates with findings for the human PPIP5K2 kinase domain, whereby the first-order rate constant for 5-IP_7_ phosphorylation was 22-fold greater than the rate constant for IP_6_ phosphorylation (14) and *k*_cat_/*K*_m_ for 5-IP_7_ phosphorylation was 29-fold greater than for IP_6_ phosphorylation (13). We found that a strong preference of Asp1 kinase for 5-IP_7_ versus IP_6_ as the phosphate acceptor was maintained under competitive substrate conditions in which IP_6_ was present in molar excess over 5-IP_7_. Our findings for Asp1 are consistent with the proposal by Shears and colleagues (29) that the synthetic path from IP_6_ to IP_8_ *in vivo* entails sequential conversion of IP_6_ to 5-IP_7_ by 5-kinases Kcs1/IP6K and phosphorylation of 5-IP_7_ to IP_8_ by 1-kinases Asp1/Vip1/PPIP5K.

### Full-length Asp1.

We show here that full-length Asp1 catalyzes futile cycles of 1-phosphate phosphorylation by its kinase component and 1-pyrophosphate hydrolysis by its pyrophosphatase component that result in unproductive net consumption of the ATP substrate. The H397A mutation in the pyrophosphatase active site restored net IP_8_ synthesis by full-length Asp1-H397A to nearly the same specific activity as the isolated Asp1 kinase domain. A crucial finding here, inspired by studies of PPIP5K2, was that increasing concentrations of inorganic phosphate, the product of the IPP pyrophosphatase reaction, enabled net IP_8_ synthesis *in vitro* by full-length wild-type Asp1. Significant activation of IP_8_ synthesis was evident at 25 mM phosphate, which is the reported physiological intracellular concentration of orthophosphate in budding yeast grown in phosphate-replete medium (27). Phosphate was more effective than sulfate in reviving IP_8_ synthesis by full-length Asp1. We attribute the phosphate effect to inhibition of Asp1’s confounding pyrophosphatase activity, given that the IP_7_ kinase activity of the isolated kinase domain was unaffected by up to 50 mM phosphate. Although we do not exclude the existence of other factors or metabolites that might regulate Asp1 activity *in vivo*, or of potential interdomain allostery, our findings anent phosphate *in vitro* provide a simple and plausible account of how the Asp1 can achieve net IP_8_ synthesis in the cellular milieu.

### Structure-guided mutagenesis with *in vitro* and *in vivo* readouts.

Analyses of fis sion yeast strains bearing kinase-defective *asp1* mutant alleles (*asp1*Δ or *asp1-D333A*) that have no intracellular IP_8_, and of cells with a pyrophosphatase-defective *asp1-H397A* allele that have elevated levels of IP_8_, implicate IP_8_ in a variety of physiological events (9, 10, 19, 30–32). At the transcriptional level, IP_8_ governs expression of the fission yeast phosphate regulon, such that Pho1 acid phosphatase is hyper-repressed in cells lacking IP_8_ and overexpressed in cells with elevated IP_8_ (19). Because cell surface Pho1 activity provides a quantitative gauge of the function of Asp1 kinase, we sought to correlate mutational effects on Asp1 5-IP_7_ kinase activity *in vitro* with the derepression of Pho1 *in vivo* when the mutant kinase domains were expressed in *asp1*Δ cells. Our alanine scan of the kinase, guided by the crystal structure of a human PPIP5K2 transition-state analog (14), identified Asp321, Asp333, Lys260, Arg223, and His204 as essential for Asp1 kinase activity *in vitro* and Pho1 derepression *in vivo*. The importance of these amino acids for catalysis is sensible insofar as the equivalent side chains in PPIP5K2 are those that bind the two metal ions, the inositol 1-phosphate, and the ATP phosphates in the transition state. Alanine scanning of PPIP5K2 had shown that Arg213 (Arg223 in Asp1) and Lys248 (Lys260 in Asp1) are essential for PPIP5K2’s 5-IP_7_ kinase activity (14). We found that expression of full-length wild-type Asp1 in *asp1*Δ cells was less potent than the Asp1 kinase domain in its extent of Pho1 derepression, presumably because the degree of IP_8_ accumulation *in vivo* was attenuated by the pyrophosphate domain. Consistent with this idea, we saw that alanine mutations of pyrophosphatase active site constituents His397 and Arg396 restored derepression to the same level as the kinase domain.

Further mechanistic insights into Asp1 activity and its regulation will hinge on obtaining atomic structures of the component kinase and pyrophosphatase domains, and especially the full-length bifunctional enzyme, in complexes with reactants and products at discrete steps along the respective reaction pathways.

## MATERIALS AND METHODS

### Recombinant Asp1 proteins.

pET28b-His_10_Smt3-Asp1-(1-385) plasmids encoding the Asp1 kinase domain (or alanine mutants thereof) fused to an N-terminal His_10_Smt3 tag were transformed into Escherichia coli BL21(DE3). Cultures (3.2 liters for wild-type kinase or 800 mL for alanine mutants) amplified from single transformants were grown at 37°C in Terrific Broth containing 50 μg/mL kanamycin until *A*_600_ reached 0.8, then adjusted to 2% (vol/vol) ethanol and placed on ice for 30 min. Asp1 kinase expression was induced by adding isopropyl ß-D-1-thiogalactopyranoside (IPTG) to 0.5 mM and incubating the cultures overnight at 17°C with constant shaking. Cells were harvested by centrifugation and resuspended in buffer A (50 mM Tris-HCl pH 8.0, 500 mM NaCl, 10% glycerol) containing 10 mM imidazole and one cOmplete Protease Inhibitor Cocktail tablet (Roche) at a volume of 25 mL per L of culture. All subsequent purification procedures were performed at 4°C. Cell lysis was achieved by adding lysozyme to 0.5 mg/mL and incubating for 1 h, followed by sonication to reduce viscosity. The lysate was centrifuged at 38,000g for 45 min and the supernatant was mixed with 5 mL of Ni-NTA-agarose resin (Qiagen) that had been equilibrated in buffer A with 10 mM imidazole. After 1 h of mixing on a nutator, the resin was recovered by centrifugation and washed twice with 50 mL of buffer A containing 20 mM imidazole. The washed resin was poured into a column and the bound protein was eluted with 250 mM imidazole in buffer A. The elution of His_10_Smt3-Asp1-(1-385) protein was monitored by SDS-PAGE. The His_10_Smt3 tag was cleaved by treatment with Ulp1 protease (100 μg Ulp1 per L of bacterial culture) during overnight dialysis against buffer A with 20 mM imidazole. Asp1-(1-385) proteins were separated from the His_10_Smt3 tag by a second round of Ni-affinity chromatography, during which Asp1-(1-385) proteins were recovered in the flow-through fraction. Tag-free Asp1-(1-385) was concentrated to a volume of 5 mL by centrifugal ultrafiltration and then applied to a Hiload Superdex 200 pg 16/600 column (Cytiva Life Sciences) equilibrated in buffer B (30 mM HEPES, pH 6.8, 150 mM NaCl, 10% glycerol). The peak Superdex fraction of each Asp1-(1-385) preparation was concentrated by centrifugal ultrafiltration and stored at −80°C. Protein concentrations were determined by using the Bio-Rad dye reagent with BSA as the standard.

pET28b-His_10_Smt3-Asp1 plasmids encoding the full-length Asp1 protein (or alanine mutants thereof) fused to an N-terminal His_10_Smt3 tag were transformed into Escherichia coli BL21(DE3). IPTG induction of Asp1 expression and purification of Asp1 from soluble bacterial lysates was performed as described above for the Asp1 kinase domain.

### TLC assay of Asp1 kinase and ATPase activity.

Reaction mixtures containing 30 mM Bis-Tris (pH 6.2), 50 mM NaCl, MgCl_2_, [γ^32^P]ATP, IP_6_ (phytic acid; Sigma P-8810-10G, lot BCBZ7573) or IP_7_ (synthesized as described in 21–23), and Asp1-(1-385) at concentrations specified in the figure legends were incubated at 37°C. Reactions were initiated by addition of Asp1 and quenched at the times specified by adjustment to 25 mM EDTA. Aliquots (2 μL) were applied to a PEI-cellulose TLC plate (Millipore-Sigma), and the products were resolved by ascending TLC with 1.7 M ammonium sulfate as the mobile phase. The radiolabeled ATP substrate and P_i_ and IPP products were visualized by autoradiography or visualized and quantified by scanning the TLC plate with a Typhoon FLA7000 imager and ImageQuant-TL software.

### PAGE assay of Asp1 kinase activity.

Reaction mixtures (20 μL) containing 30 mM Bis-Tris (pH 6.2), 50 mM NaCl, MgCl_2_, ATP, and IP_6_ or IP_7_ as specified in the figure legends were incubated at 37°C. Reactions were terminated at the times specified by adjustment to 25 mM EDTA. The samples were mixed with an equal volume of 2× Orange G loading buffer (10 mM Tris-HCl, pH 7.0, 1 mM EDTA, 30% glycerol, 0.1% Orange G dye) and then analyzed by electrophoresis (at 4°C at 8 W constant power) through a 20-cm 36% polyacrylamide gel containing 80 mM Tris-borate, pH 8.3, 1 mM EDTA until the Orange G dye reached 2/3 of the length of the gel. The gel was briefly washed with water and then stained with a solution of 0.1% Toluidine blue (Sigma), 20% methanol, 2% glycerol, followed by destaining in 20% methanol.

### pTIN-based expression of Asp1 in *asp1*Δ fission yeast.

pTIN plasmids (24) encoding the Asp1 kinase domain (aa 1–385) or alanine mutants thereof were transfected by the lithium acetate method into S. pombe *asp1*Δ cells. Control transfection was performed with the empty pTIN vector. Transformants were selected on Leu^−^ *e*nhanced *P*ombe *M*inimal *G*lutamate (ePMG) 2% agar medium with 15 μM thiamine. The recipe for 1 L of ePMG liquid medium contains the following ingredients: potassium hydrogen phthalate (3.0 g); anhydrous sodium phosphate dibasic (1.66 g); anhydrous sodium phosphate monobasic (0.46 g); glucose (20 g); adenine (0.25 g); uracil (0.25 g); glutamic acid (3.75 g); histidine (0.25 g); lysine (0.25 g); 1,000× vitamins (1 mL); 10,000× minerals (0.1 mL); 50× salts (20 mL); and Leu^−^ amino acid mix (2.5 g). The components of the vitamin, mineral, and salt stocks are as defined previously (33). The Leu^−^ amino acid mix is composed of alanine (2.8 g), arginine (1.3 g), asparagine (0.5 g), aspartic acid (2.65 g), cysteine (0.10 g), glutamine (0.1 g), glutamic acid (4.70 g), glycine (1.50 g), histidine (0.65 g), isoleucine (1.5 g), lysine (2.3 g) methionine (0.4 g), phenylalanine (1.3 g), proline (1.0 g), serine (0.8 g), threonine (0.8 g), tryptophan (0.25 g), tyrosine (0.60 g), and valine (1.75 g). The pH of ePMG is adjusted to 5.6 as needed by addition of NaOH.

### Acid phosphatase activity.

S. pombe *Δasp1* cells bearing pTIN plasmids were grown at 30°C in Leu^−^ ePMG liquid medium with 15 μM thiamine. Aliquots of exponentially growing cultures were harvested, washed with water, and resuspended in water. To quantify acid phosphatase activity, reaction mixtures (200 μL) containing 100 mM sodium acetate (pH 4.2), 10 mM *p*-nitrophenylphosphate, and cells (ranging from 0.01 to 0.1 *A*_600_ units) were incubated for 5 min at 30°C. The reactions were quenched by addition of 1 mL of 1 M sodium carbonate, the cells were removed by centrifugation, and the absorbance of the supernatant at 410 nm was measured. Acid phosphatase activity is expressed as the ratio of *A*_410_ (*p*-nitrophenol production) to *A*_600_ (cells). The data are averages (±SEM) of at least three assays using cells from three independent cultures.

### Asp1 antibody.

Rabbit immunization with purified Asp1-(1–364) and preparation of antiserum were performed by Pocono Hills Rabbit Farm and Laboratory (Canadensis, PA) according to their Mighty Quick Protocol. Anti-Asp1 antibody was purified from rabbit serum by affinity chromatography as follows. Purified Asp1 kinase (4.5 mg) was dialyzed against coupling buffer (100 mM HEPES, pH 6.5, 500 mM NaCl, 5% glycerol) and then coupled to 4 mL of Affigel-10 resin (Bio-Rad) during overnight incubation at 4°C. The resin was washed serially with 100 mM Tris-HCl, pH 7.5; 200 mM glycine, pH 2.6; 1 M Tris-HCl, pH 7.5; and 20 mM Tris-HCl, pH 7.5, 150 mM NaCl. Asp1 kinase-coupled resin was then mixed with 8 mL of rabbit immune serum (adjusted to 10 mM Tris-HCl, pH 7.5) for 2 h at room temperature on a nutator. The resin was poured into a column and washed thoroughly with 10 mM Tris-HCl, pH 7.5, 150 mM NaCl until no further protein eluted, as gauged by Bio-Rad dye-binding assay of wash fractions. Bound antibodies were eluted with 200 mM glycine, pH 2.6 while collecting fractions (1 mL) in tubes containing 100 μL of 1 M Tris-HCl, pH 7.5 to adjust the pH. Protein-containing eluate fractions were pooled, dialyzed against buffer containing 10 mM Tris-HCl, pH 7.5, 150 mM NaCl, and concentrated to 0.36 mg/mL.

### Western blotting.

S. pombe *asp1***Δ** cells bearing pTIN plasmids were grown at 30°C in Leu^−^ ePMG liquid medium with 15 μM thiamine until *A*_600_ reached 0.6 to 0.9. Aliquots (8 *A*_600_ units) of cells were collected by centrifugation and resuspended in 200 μL 20% trichloroacetic acid, then supplemented with 0.7 g of 0.5 mm Zirconia beads (Biospec) and subjected to six 30-s cycles of treatment with a FastPrep-24 bead-beater (MP biomedical) at 6.5 m/s. Total acid-insoluble protein was recovered by centrifugation. The pellets were washed twice with ethanol, then air dried and resuspended in 300 μL 0.5 M Tris-HCl (pH 8.0). The samples were adjusted to 2% SDS, 10% glycerol, 10% β-mercaptoethanol and heated at 95°C for 5 min. Cell debris and insoluble material were removed by centrifugation. Aliquots of supernatant proteins (representative of 0.36 *A*_600_ units of cells) were resolved by electrophoresis through 8% polyacrylamide gels containing 0.1% SDS. Gel contents were transferred to a polyvinylidene difluoride (PVDF) membrane (Invitrogen). The blots were probed with affinity-purified rabbit polyclonal anti-Asp1 protein. Parallel blots were probed with anti-Spt5 antibody as a loading control. Immune complexes were detected using horseradish peroxidase-linked anti-rabbit IgG (Cytiva NA934V) and an ECL (enhanced chemiluminescence) Western system (Cytiva) and visualized with an ImageQuant 800 apparatus (Amersham).
